# A whole exome sequencing study to identify rare variants in multiplex families with alcohol use disorder

**DOI:** 10.3389/fpsyt.2023.1216493

**Published:** 2023-10-17

**Authors:** Shirley Y. Hill, Joseph Hostyk

**Affiliations:** ^1^Department of Psychiatry, Psychology and Human Genetics, University of Pittsburgh, Pittsburgh, PA, United States; ^2^Institute for Genomic Medicine, Columbia University, New York, NY, United States

**Keywords:** rare variants, alcohol use disorder, multiplex families, substance use disorder, exome

## Abstract

**Background:**

Alcohol use disorder (AUD) runs in families and is accompanied by genetic variation. Some families exhibit an extreme susceptibility in which multiple cases are found and often with an early onset of the disorder. Large scale genome-wide association studies have identified several genes with impressive statistical probabilities. Most of these genes are common variants. Our goal was to perform exome sequencing in families characterized by multiple cases (multiplex families) to determine if rare variants might be segregating with disease status.

**Methods:**

A case-control approach was used to leverage the power of a large control sample of unrelated individuals (*N* = 8,983) with exome sequencing [Institute for Genomic Medicine (IGM)], for comparison with probands with AUD (*N* = 53) from families selected for AUD multiplex status. The probands were sequenced at IGM using similar protocols to those used for the archival controls. Specifically, the presence of a same-sex pair of adult siblings with AUD was the minimal criteria for inclusion. Using a gene-based collapsing analysis strategy, a search for qualifying variants within the sequence data was undertaken to identify ultra-rare non-synonymous variants.

**Results:**

We searched 18,666 protein coding genes to identify an excess of rare deleterious genetic variation using whole exome sequence data in the 53 AUD individuals from a total of 282 family members. To complete a case/control analysis of unrelated individuals, probands were compared to unrelated controls. Case enrichment for 16 genes with significance at 10^–4^ and one at 10^–5^ are plausible candidates for follow-up studies. Six genes were ultra rare [minor allele frequency (MAF) of 0.0005]: *CDSN*, *CHRNA9*, *IFT43*, *TLR6*, *SELENBP1*, and *GMPPB*. Eight genes with MAF of 0.001: *ZNF514*, *OXGR1*, *DIEXF*, *TMX4*, *MTBP*, *PON2*, *CRHBP*, and *ANKRD46* were identified along with three protein-truncating variants associated with loss-of-function: *AGTRAP*, *ANKRD46*, and *PPA1*. Using an ancestry filtered control group (*N* = 2,814), nine genes were found; three were also significant in the comparison to the larger control group including *CHRNA9* previously implicated in alcohol and nicotine dependence.

**Conclusion:**

This study implicates ultra-rare loss-of-function genes in AUD cases. Among the genes identified include those previously reported for nicotine and alcohol dependence (*CHRNA9* and *CRHBP*).

## Background

The role of rare genetic variation in the susceptibility to alcohol use disorders (AUDs) has been investigated in fewer studies than those that have addressed common variants. Genome-wide association studies (GWAS) have predominated in studies of generic vulnerability to AUDs. These studies have pointed to significantly associated loci that may hold promise for understanding AUD etiology and suggest alternatives for medication development. With increasing sample sizes, many GWAS signals reported for complex phenotypes have explained a smaller effect on risk (1). The proliferation of next generation sequencing (NGS) studies has enabled investigation into the possible contribution of rare variants that can be identified through NGS.

## Materials and methods

### Sample description

Multiplex alcohol dependence families were identified based on the presence of a same-sex sibling pair, each with a diagnosis of alcohol dependence by DSM-III criteria, the prevailing DSM at study initiation. One member of the proband pair was recruited from a treatment facility in the Pittsburgh area. Those providing permission to contact relatives for preliminary screening were provisionally included. Following screening, only families who were free of other psychiatric comorbidity (other than alcohol dependence) in the first and second-degree relatives of the proband pair were included. Families were excluded if recurrent major depression, bipolar disorder, schizophrenia, or a primary substance use disorder (SUD) other than alcohol dependence was present in either the proband pair of adult alcohol dependent individuals or their first degree relatives. Alcohol dependence was required to have occurred at least 1 year before the onset of the SUD in all individuals studied. Accordingly, those cases with a comorbid SUD present were considered to be secondary disorders.

All participants in the family study (the same sex proband pair of siblings, other siblings, and parents of the siblings) including those with sequencing were administered a structured, in-person, psychiatric interview [Diagnostic Interview Schedule (DIS)] (2) by a trained Masters-level clinician, allowing for a diagnosis of alcohol dependence by DSM-III (3) (the diagnostic system in place when the study was initiated) to be determined. Each participant was administered questions necessary to determine whether the participant met Feighner criteria (4) for alcohol dependence. The Feighner criteria diagnosis requires at least one symptom in three out of four symptom categories. The categories were: Category 1 – medical consequences (e.g., withdrawal symptoms or health problems); Category 2 – attempt to control drinking (e.g., limit use to certain times of the day, drinking before breakfast, and use of non-beverage alcohol such as mouth wash); Category 3 – legal/social problems (e.g., arrests, fights, and DUI); and Category 4 – the participant’s report of excessive drinking (e.g., family/friends object to participant’s drinking and the participant felt guilty about their own drinking). The earliest age the participant had at least one symptom in each category was determined. The age at which three problem categories were positive was considered to be the age of alcohol dependent use.

### Validity of clinical data

The interview data was supplemented with an open-ended clinical interview by a second clinician in order to provide reliability of the DIS and Feighner criteria interviews. A best-estimate diagnosis of alcohol dependence was made using the information from the DIS, the Feighner criteria, the second open-ended interview, and any family history information provided by the participating relatives. For the present analysis, DIS items were rescored to be consistent with DSM-V and the average number of symptoms accumulated to provide current validity of the diagnoses obtained using the earlier DSM-III nomenclature.

### Demographic characteristics of AUD cases (*N* = 53) analyzed

Although each family included a proband pair with an AUD, for the purpose of the present analysis, only one member of the pair was included to insure that unrelated cases could be compared with unrelated controls. The demographic characteristics for these individuals are presented here. The probands included 26 women and 27 men. Self-reported ancestry was 94% Caucasian and 6% African-American. They averaged 34.3 ± 6.3 years at interview.

### Demographic characteristics of controls

Based on self-reported ancestry, the full control group of 8,893 individuals consisted of 58.1% Caucasian, 14.3% African-American, 12.1% Hispanic, with East Asian, Middle Eastern, and South Asian representing 0.7% of the total. Another 14.7% were of unknown ancestry (Table 1).

**TABLE 1:** Ancestry of IGM controls used in comparison analyses with 53 AUD cases.

	African-American	Caucasian	East Asian	Hispanic	Middle Eastern	South Asian	Unknown	Total ethnicity
Healthy controls	990	588	0	712	1	0	539	2,830
Epilepsy patients	129	2,224	0	97	10	4	186	2,650
Healthy family member	166	2,407	1	282	42	5	600	3,503
Total	1,285	5,219	1	1,091	53	9	1,325	8,983

### Exome sequencing and bioinformatic processing

Whole exome sequencing of blood-extracted DNA was performed at the Institute for Genomic Medicine (IGM) at Columbia University for 282 individuals from multiplex AUD families along with IGM control subjects using the same bioinformatic pipeline. Our goal was to compare variants observed in one member of 53 proband pairs from separate families to a large control sample maintained at IGM. Sequencing was performed using the SureSelect Human All Exon (65 MB; Agilent Technologies, Santa Clara, CA, USA) or the NimbleGen SeqCap EZ version 2.0 or 3.0 exome enrichment kit (Roche NimbleGen, Madison, WI, USA) on HiSeq 2000 or 2500 sequencers (Illumina, San Diego, CA, USA) according to standard protocols. The IGM pipeline maintained a goal of achieving 10-fold sequencing coverage. The quality of sequencing was monitored using software designed to filter the raw sequencing data. Data were excluded based on several considerations including the presence of duplicate reads, and being among a list of known sequencing artifacts. Data were included only if they were among the consensus coding sequence public transcripts (CDDS Release 14). Raw sequence data was taken in as an Illumina lane level fastq files along with the reference genome (Human Reference Genome-NCBI Build 37) to which it is aligned using the Burrows-Wheeler Alignment (BWA) tool. The new data that was generated contained the reads and the genetic location resulting in Sequence Alignment and Map (SAM) files that were then processed as a Binary Alignment and Map (BAM) files for variant calling. Identification of the variants was performed with GATK software with analyses performed using the Analysis Tool for Annotated Variants (ATAV).

We determined both the percentage of case subjects and the percentage of control subjects who had at least 10-fold coverage at each site. At least 10-fold sequencing read coverage was achieved for ≥95% of the megabase pairs (Mbp) of the consensus coding sequence (CCDS; Release 14) for the case and control subjects. The percentage of cases and controls meeting this criterion did not differ significantly insuring that results were not influenced by differential coverage in the CCDS sequence.

Individual CCDS sites were excluded from analysis if the absolute difference in percentages of case subjects compared with control subjects who achieved at least 10-fold coverage differed exceeded a predetermined threshold (<10%). Harmonization of coverage between cases and controls is an important step. Where coverage is highly imbalanced, the less well represented group can show an inability to call a particular variant. This can lead to an enrichment bias toward the better represented group (5). All collapsing tests were then performed on the pruned 30.67 Mbp of CCDS sites. This insured that both cases and controls had similar opportunity to call variants. Absence of preferential inflation of background variation was further confirmed by conducting an exome-wide tally of rare autosomal synonymous (i.e., presumed neutral) variants per individual. This tally did not find a significant difference between the case and control groups. Autosomal read depth and the sequencing coverage were consistent between the cases and control samples with no significant difference being seen.

### Statistical analysis

The search for genes potentially influencing risk for AUD was implemented using genetic collapsing tests (5). We used the conventional gene based collapsing method in which the protein coding boundaries of the gene is the unit of analysis and the criteria for comparing cases and controls is based on whether there is at least one qualifying variant in the gene. This method differs from rare variant burden tests which define a genetic region and then aggregate the information within the defined region to describe the summary dose or burden. Because genetic relatedness can distort the contribution of any particular variant to the test statistic, we first pruned our sample of 282 members of densely affected multiplex families to include only one case per family resulting in 53 cases. We chose one of the members of the proband pair that had been identified through participation in a substance use treatment program and who had led us to the remaining family members. We focused our analyses on CCDS protein-coding sites with minimal variability in coverage between the case and control populations. We analyzed qualifying variants, a term originally introduced to refer to the subset of genetic variations within the sequence data (6) that meets specific population allele frequency and predicted variant effect criteria. We defined nine different qualifying variant models in all (Table 2) for additional analysis to determine if deleterious variants might be found. The primary model of interest was one in which a search for “ultra-rare” non-synonymous variants was conducted in order to capture a category of genetic variation expected to be most enriched for variants of high effect. Ultra-rare variants were identified using an external sources (Exome Variant Server and Exome Aggregation Consortium release 0.3) (7), and sequence data from our combined case and control test populations to find variants with a minor allele frequency (MAF) of less than 0.05%. Qualifying variants were restricted to indels and single nucleotide variants annotated as having either a loss-of-function (LoF) effect, an inframe indel, or a “probably damaging” missense prediction by Polymorphism Phenotyping version 2 (PolyPhen, HumDiv^1^) (8). These analyses relied on the predicted effects of the LoF and missense annotated variants whose functions have not been individually confirmed in the laboratory. We subsequently performed analyses of CCDS genes using the nine alternative qualifying variant models as defined in Table 2, including an autosomal recessive model and a synonymous variant negative control model.

**TABLE 2:** Nine analyses completed based on differing genetic models and definitions of qualifying variants.

	Synonymous	Ultra-rare	Rare damaging REVEL	Rare damaging PolyPhen	Flexible REVEL	Flexible PolyPhen	Flexible no intolerance score	PTV	Recessive autosomal
Inheritance	Dominant	Dominant	Dominant	Dominant	Dominant	Dominant	Dominant	Dominant	Comp-het
Function	Synonymous	All functional	All functional	All functional	All functional	All functional	All functional	PTV loss of function	All functional
REVEL	None	None	>0.5	None	>0.5	None	None	None	None
Polyphen	None	Damaging	None	Damaging	None	Damaging	None	None	None
Leave one out allele frequency	0.0005	0.0005	0.0005	0.0005	0.001	0.001	0.001	0.001	0.01
gnomAD and ExAC^1^ allele frequency	0	0	0.00005	0.00005	0.001	0.001	0.001	0.001	0.01

^1^Exome Aggregation Consortium (7) browser is no longer available. Data is available in gnomAD browser. - means compound heterozygote.

For each of the nine models, we tested the list of 18,666 CCDS genes. For each gene, an indicator variable (1/0 states) was assigned to each individual on the basis of presence of at least one qualifying variant in the gene (state 1) or no qualifying variants in that gene (state 0). A two-tailed Fisher’s exact test (FET) was then performed for each gene to compare the rate of case subjects carrying a qualifying variant compared with the rate of control subjects. For our study-wide multiplicity adjusted significance threshold, a Bonferroni correction was made using the number of genes tested across the non-synonymous models. The Bonferroni threshold for a single model is 2.67 × 10^–6^. Because our primary interest was in ultra-rare variants this threshold is appropriate for the primary analysis. For the complete analysis of the nine models, the appropriate threshold is 2.97 × 10^–7^. We did not correct for the synonymous (negative control) model due to differing sampling rates between cases and controls for genes on the X chromosome. Collapsing analyses were performed using an in-house package, ATAV.^2^ Additional binomial analyses, logistic regression analyses, and FETs were completed using the “stats” package in R version 3.2.2 (R Foundation for Statistical Computing, Vienna, Austria).

## Results

### Clinical characteristics of probands

All probands met DSM-III criteria for alcohol dependence, the diagnostic scheme in place at the time of the participant ascertainment. Additionally, they met Feighner criteria for alcohol dependence, and DSM-V criteria for AUD (Table 3). Using individual responses to the DIS interview retained in file, all probands were diagnosed with DSM-V criteria. All probands met DSM-V criteria (Table 3). Eight of the 11 conceptual areas included in DSM-V were available for scoring using responses to questions that mapped to DSM-V concepts. The mean and standard deviation for the sample was 5.88 ± 1.01 symptoms, indicating a severe form of AUD overall.

**TABLE 3:** Clinical characteristics of probands.

	Disorder present		Disorder absent	
	***N***	**(%)**	***N***	**(%)**
DSM-III alcohol dependence	53	100	0	0
Feighner criteria – alcohol dependence	53	100	0	0
Age of onset first problem group	17.8 ± 6.4			
Age of onset 3 problem groups	23.3 ± 7.6			
DSM-III drug dependence	31	58.5	22	41.5
DSM-III anxiety disorder	3	5.7	50	94.3
DSM-III MDD	11	20.8	42	79.2
DSM-III schizophrenia	0	0	53	100
Ever a smoker	36	67.9	16	30.2^a^
**DSM-V^b^**	**No disorder**	**Mild**	**Moderate**	**Severe**
Number and% by category	0 (0)	1 (1.9)	14 (26.4)	38 (71.7)

^a^Data for one case was missing. ^b^Individual responses to Feighner criteria questions were retained in file. These questions mapped to 8 of the 11 categories currently included in DSM-V for AUD. DSM-V utilizes the presence of 2–3 categories as mild AUD, 4–5 as moderate, and 6 or more as severe. See American Psychiatric Association (50).

### Gene set enrichment

We searched 18,666 protein coding genes for a statistically significant excess of rare deleterious variation in 282 members of multiplex AUD families. The present report is based on a comparison of 53 unrelated probands (one per family) from these families in comparison to a data set of 8,893 unrelated controls similarly sequenced at IGM. Our results are presented in three sections: (1) primary analyses using all available controls (8,893); (2) secondary analyses using ancestry filtered controls (*N* = 2,814) providing the closest match to the cases with AUD; and (3) analyses directed by a previous search of genes identified in a GWAS analysis conducted by Peng et al. (9) that included analysis for rare variants.

#### Primary analyses

##### Ultra-rare variants

**FIGURE 1:**
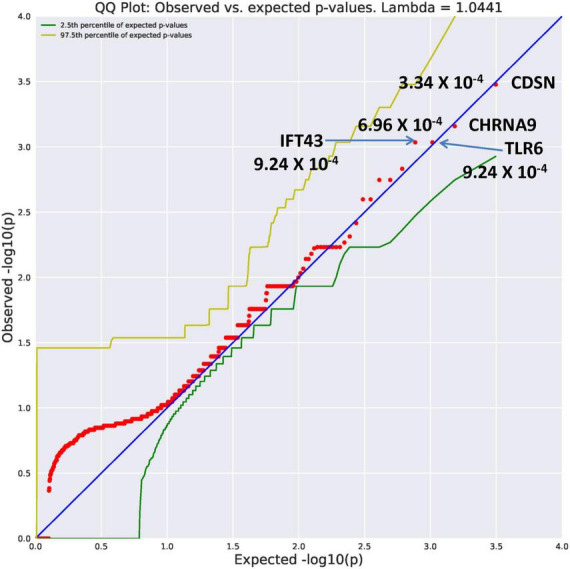
A search of megabase pairs of the consensus coding sequence (CCDS) for 18,666 genes was conducted to identify rare variants differing between alcohol use disorder (AUD) probands from high-density AUD families and controls. A quantile-quantile (QQ) plot was used to evaluate results of the permutation expected probability distribution (5). Lambda values were calculated to determine if exome-wide inflation existed and found to be absent further indicating valid statistical results. This figure illustrates four genes with ultra rare minor allele frequency (MAF) of 0.0005 with significance levels reaching 10^– 4^. The observed versus expected values are plotted.

Ultra-rare variants had an allele frequency of 0.0005 (Table 2). In comparison to the unrelated controls, the AUD probands displayed an increase in genes identified with an ultra-rare qualifying model. The top genes (Figure 1) were *CDSN* (OR = 117.39, *p* = 3.34 × 10^–4^), *CHRNA9* (OR = 70.42, *p* = 6.96 × 10^–4)^, *IFT43* (OR = 58.67, *p* = 9.24 × 10^–4^), and *TLR6* (OR = 58.67, *p* = 9.24 × 10^–4)^. Using a Bonferroni threshold of 2.67 × 10^–6^ for multiple testing (0.05/18,666 genes) the top genes did not reach study-wide statistical significance. A complete list of all genes with variants identified as ultra-rare can be seen in Supplementary Table 1. Two of the genes, *CDSN* and *TLR6* mediate immune functioning. One gene, *IFT43*, is involved in intraflagellar transport; another, *CHRNA9* is a member of the nicotine acetylcholine receptor family. Similarly, multiple testing correction indicates these genes listed in Supplementary Table 1 would not reach study-wide significance. The function of these genes appears to suggest potential value for follow-up.

The *CDSN* gene is part of the major histocompatibility complex on Chromosome 6 (10). The protein encoded by *TLR6* is part of the toll-like receptor family influencing the production of cytokines involved in immune response (11). These genes have not been directly related to brain function. However, the relationship between immune signaling in the brain and development of AUDs is an active area of research (12). The *IFT43* gene encodes a subunit of the intraflagellar transport complex A and plays an important role in cilia assembly and maintenance (13). The *CHRNA9* gene is a member of the ligand-gated ionic channel family. This gene has been identified in three previous investigations from different data sets as influential in alcohol dependence (14), nicotine dependence (14, 15), and in drug dependence (16).

##### Ultra-rare damaging genes

**FIGURE 2:**
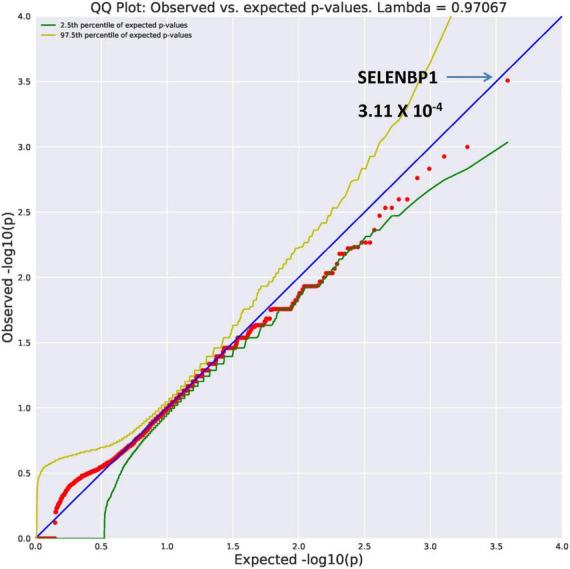
Search of the CCDS for the 18,666 genes contrasting AUD probands and controls revealed two ultra rare genes (MAF = 0.0005) that also qualified as potentially damaging. The SELENBP1 gene found with the rare damaging PolyPhen2 algorithm is shown here.

*SELENBP1* (OR = 26.89, *p* = 3.11 × 10^–4)^ and *GMPPB* (OR = 26.89, *p* = 3.11 × 10^–4^) were found to be ultra-rare. Results from REVEL analysis for SELENBP1 is seen in Figure 2. While variation in four genes (Figure 1) are ultra-rare, the *SELENBP1* and *GMPPB* genes are ultra-rare but also considered potentially damaging as revealed by PolyPhen and REVEL algorithms, respectively. The two genes have known functions. *GMPPB* is thought to encode a GDP-mannose pyrophosphorylase (17). Mutations in this gene have been found in association with limb-girdle muscular dystrophy (18). Selenium is an essential nutrient with its utilization dependent on actions of the *SELENBP1* gene which encodes a member of the selenium-binding protein relevant to preventions of some cancers and neurological diseases (19).

##### Rare variants

**FIGURE 3:**
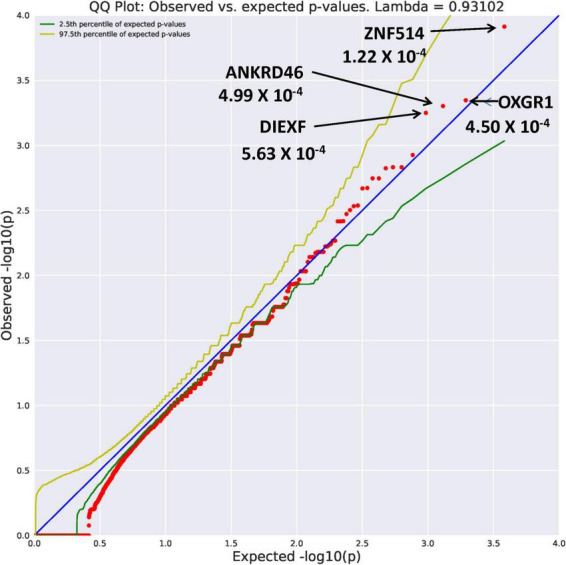
Search of the CCDS for the 18,666 genes contrasting AUD probands and controls using Flexible REVEL algorithm revealed four rare genes (MAF = 0.001) with Fisher’s exact test probability reaching 10^– 4^.

**FIGURE 4:**
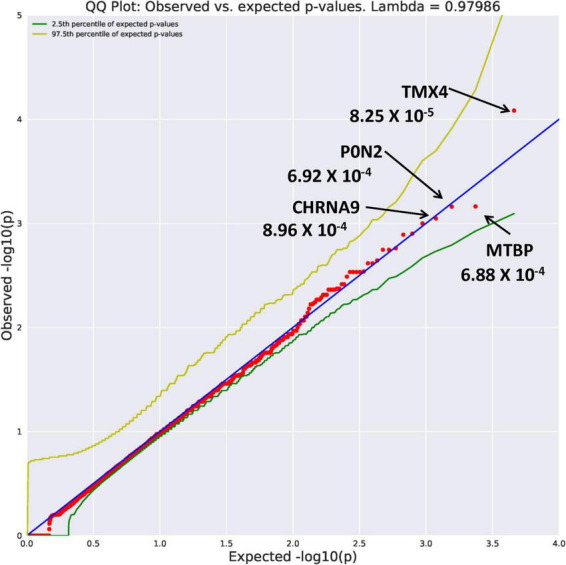
Search of the CCDS for the 18,666 genes contrasting AUD probands and controls using Flexible PolyPhen2 algorithm revealed four rare genes (MAF = 0.001) with Fisher’s exact test probability reaching 10^– 4^.

Eight genes with an allele frequency of 0.001 were our top genes in a comparison between the unrelated AUD probands and unrelated controls. These are *ZNF514* (OR = 38.44, *p* = 1.22 × 10^–4^), *OXGR1* (OR = 23.37, *p* = 4.5 × 10^–4^), *DIEXF* (OR = 21.5, *p* = 5.63 × 10^–4^), and *ANKRD46* (OR = 88.03, *p* = 3.11 × 10^–4^) (Figure 3), and *TMX4* (OR = 44.86, *p* = 8.25 × 10^–5^), MTBP (OR = 11.2, *p* = 6.88 × 10^–4^), and *PON2* (OR = 19.9, *p* = 6.92 × 10^–4^) (Figure 4). Additionally, *CRHBP* (OR = 20.67, *p* = 6.25 × 10^–4^) differed between probands and controls. The complete list can be seen in Supplementary Table 1. A brief description of the function of these genes follows. Zinc Finger protein 514 (*ZNF514*) is predicted to be involved in regulation of transcription by RNApolymerase II (20). The oxoglutarate receptor 1 (*OXGR1*) gene encodes a G protein receptor, GPCR, and is implicated in many inflammatory disorders (21). The digestive organ expansion factor (*DIEFX*) gene is involved in RNA binding activity and is a possible negative regulator of *P53* in human cancers and appears to be an essential murine development gene (22). The Thioredoxin Related Transmembrane 4 (*TMX4*) gene encodes a member of the disulfide isomerase family of endoplasmic reticulum proteins (23). The *MTBP* gene may be involved in tumor formation (24) and has been reported to be over expressed in triple negative breast cancer (25). The *PON2* gene is a member of the paraoxonase gene family currently thought to include three members all of which are located on the long arm of Chromosome 7 (26). Variation in the *PON2* gene has been associated with vascular disease, diabetes phenotypes and Amyotrophic Lateral Sclerosis (27). The Corticotrophin Releasing Hormone Binding Protein (*CRHBP*) gene is a protein coding gene which stimulates the synthesis and secretion of proopiomelanocortin-derived peptides (28).

Variations in stress genes *CRHR1* and *CRHBP* that are part of the HPA axis have been reported to influence alcohol drinking in rats and mice and show associations in clinical studies of AUD (29–31). Also, an association between a genetic variant in the *CRHBP* gene and reduction in cocaine abuse in methadone maintained participants has been reported (32). Additionally, an interaction between life stress and variation in *CRHBP* has been reported as influential in heroin dependence relapse (33). Also, the presence of AUD as a comorbid condition in schizophrenic individuals has been associated with variation in this gene (34). Recently, we reported an association for the *CRHR1* gene and AUD in individuals with multiplex familial loading for AUD (35). Overall, associations between these eight rare genes with MAF of 0.001 or less and alcohol or drug use disorders have previously been reported infrequently though *CRHR1* and *CHRA9* have support from multiple studies.

##### Protein truncating variants

**FIGURE 5:**
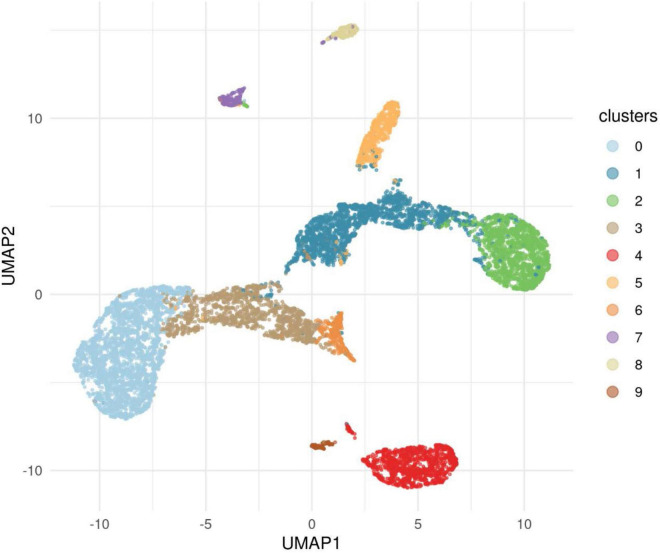
This figure illustrates the results of applying the algorithm Uniform Manifold Approximations and Projection (UMAP) to the data for cases and controls to reveal clusters. The 10 clusters revealed showed that Cluster 0, one of the European clusters, was represented in both cases and controls. This cluster provided a comparison with significant differences found for nine genes.

Three genes were identified as having protein truncating variants associated with loss of function that differed in frequency between the AUD probands and the controls. These are: *AGTRAP* (OR = 352.24, *p* = 1.01 × 10^–4^), *ANKRD46* (OR = 176.1, *p* = 2.01 × 10^–4^), and *PPA1* (OR = 58.61, *p* = 9.24 × 10^–4^) (Figure 5). The *AGTRAP* gene product interacts with angiotensin II type 1 receptor providing a negative feedback loop in the regulation of angiotensin II. Known diseases associated with this gene include essential hypertension (36). *ANKRD46* encodes a protein containing multiple Ankyrin repeats and is involved in a variety of cellular processes (37). The *PPA1* gene encodes a protein that is involved in phosphate metabolism in cells (38).

#### Secondary analyses-ancestry filtered controls

The primary analysis included individuals who were affected with AUD but were also one member of a pair of affected probands that provided the selection criterion for inclusion of the family. The primary analysis compared 53 cases to 8,893 controls from the IGM data base. A secondary analysis was performed in which the ancestry of cases and controls were analyzed to uncover clusters of ancestry that could be utilized to compare cases with AUD and controls with similar ancestry. In addition to the 53 proband cases, 5 additional cases were sequenced from individuals affected with AUD (one per family) but who were not from the identified proband pair. These 58 cases were analyzed along with all available IGM controls to determine clusters of ancestry. This analysis used an algorithm, Uniform Manifold Approximation and Projection (UMAP) to provide clusters within the data sets. This analysis revealed 10 ancestry clusters: African, East Asian, three European clusters, two Latino clusters, two Middle Eastern clusters and one South Asian (Figure 5). Subsequent analyses comparing cases and controls with similar ancestry utilized one of the European groups allowing comparison of 52 cases and 2,814 controls (Table 4). From this comparison, nine genes showed statistically significant differences at *p* = 10^–4^: *ADCY10*, *KIAA0513*, *MTBP*, *RUFY1*, *ZHX3*, *GPI*, *AGTRAP*, *COLBA2*, and *DAGLB*. A complete list is seen in Supplementary Table 2.

**TABLE 4:** Ancestry clusters for 58 AUD cases and IGM controls.

Cluster number	Ancestry	Number of cases	Number of controls*
Cluster 0	European 1	52	2,814
Cluster 1	Latino 1	NA	2,152
Cluster 2	African 1	2	1,642
Cluster 3	European 2	4	1,446
Cluster 4	Middle Eastern 1	NA	1,212
Cluster 5	Latino 2	NA	731
Cluster 6	Middle Eastern 2	NA	330
Cluster 7	South Asian 1	NA	241
Cluster 8	East Asian 1	NA	208
Cluster 9	European 3	NA	132

*Additional IGM controls were identified for this ancestry filtered analysis.

The protein encoded by Adenylate Cyclase 10 (*ADCY10*) belongs to a class of cyclases that are insensitive to G protein. The protein catalyzes the formation of the signaling molecule cAMP (39). Case/control comparison revealed an OR = 112.52, *p* = 9.58 × 10^–4^. Variation within the *KIAA0513* gene also differed between cases and controls with an OR = 112.52, *p* = 9.58 × 10^–4^. This gene produces a novel signaling molecule involved in neuroplasticity and apoptosis (40). The *MTBP* gene with an associated MDM2 binding protein, is often overexpressed in human malignancies including triple negative breast cancer (25) and is involved in suppression of invasive behavior of hepatocellular carcinoma (41). Case/control comparison in our sample found an OR = 112.52, *p* = 9.58 × 10^–4^. The *RUFY1* gene is one of a family of effector proteins involved in intracellular trafficking with dysfunction associated with severe pathology (42). Case/control comparison in our sample found an OR = 112.52, *p* = 9.58 × 10^–4^. A significant difference between cases and controls was also found for the *ZHX3* gene (OR = 112.52, *p* = 9.58 × 10^–4^). This gene belongs to the zinc-fingers and homeobox family which act as transcriptional repressors binding with promoter regions to regulate transcription of target genes and is frequently involved in human diseases (43). The Glucose-6-phosphate isomerase (*GPI*) gene plays an important role in glycolysis and gluconeogenesis and has been identified as a biomarker for lung adenocarcinoma (44). Case/control comparison in our sample found an OR = 112.52, *p* = 9.58 × 10^–4^. The *AGTRAP* gene which provides a negative feedback loop in the regulation of angiotensin II showed significance in the comparison between probands and the larger control sample but also was significant in the comparison with the European ancestry controls (OR = 112.52, *p* = 9.58 × 10^–4^). The Collagen Type VIII Alpha 2 (*COLBA2*) gene showed a greater frequency in AUD cases in comparison to ancestry filtered controls (OR = 112.52, *p* = 9.58 × 10^–4^). This gene product is a major component of the basement membrane of the corneal epithelium with variants associated with corneal dystrophy (27). The Diacylglycerol Lipase Beta (*DAGLB*) gene catalyzes the hydrolysis of arachidonic acid esterified diacylglycerols to produce the principal endocannabinoid (45). This gene variant was significantly more frequent in the AUD cases than in controls (OR = 112.52, *p* = 9.58 × 10^–4^).

##### Analyses directed at previous gene identification

Peng et al. (9) found GWAS hits from their analyses of two cohorts, American-Indian (AI) and European-American (EA). Analyses based on the MAF provided information on whether the signals found qualified as rare variants. The signals for 48 genes reported ranged from 10^–6^ to 10^–9^ with MAF between 0.03 and 0.0005. We ran a gene set analysis using the AI and EA genes across our cases to determine if our cases exhibited an excess of rare variants in those genes. Using the nine collapsing models (Table 2) for analysis, odds ratios and FET *p*-values were calculated for the 48 genes. Across all models, four genes previously identified by Peng et al. showed significance at *p* < 0.05 in our study, *KCN2*, *NAF1*, *SLC39213*, and *PCLO*. Three additional genes identified by these investigators showed significance levels in our analyses between *p* = 0.055 and 0.08, *HMCN1*, *PRMT6*, and *PPE4C*.

## Discussion

We searched 18,666 protein coding genes to identify an excess of rare deleterious genetic variation using whole exome data sequenced at IGM for 282 AUD individuals from multiplex families. To complete a case/control analysis of unrelated individuals, one AUD case from each of the families was selected to form a group for comparison with unrelated controls similarly sequenced at IGM. Two sets of analyses were performed, one using all available IGM controls (*N* = 8,983) and one using controls of similar ancestry (*N* = 2,814). Additionally, genes previously identified in a GWAS analysis that included rare variants were tested using a gene set analysis in our sample.

### Primary analysis

Although no gene achieved genome wide significance, case enrichment for 16 genes was significant at 10^–4^ and one gene at 10^–5^ indicating they are plausible candidates for follow-up studies. A total of six gene variants qualify as ultra rare with MAF of 0.005 with two of these also considered potentially damaging: SELENBP1 and GMPPB. Four gene variants are ultra rare but not previously identified as damaging: *CDSN*, *CHRNA9*, *IFT43*, and *TLR6*. Two of the genes, *CDSN* and *TLR6* are involved in the immune response. This finding is of interest because toll-like receptors are integral components of neuroimmune adaptation with immune signaling in the brain reported to influence development of AUD (12). Extensive pre-clinical work has suggested the importance immune signaling especially for *TLR4*, a member of the toll-like receptor family, and results of clinical trials using agents that alter immune functioning to modify alcohol use (46). *IFT43* plays a role in cilia assembly and maintenance (13) but has not been reported to be related to alcohol use. *CHRNA9* is of interest because of previous reports of its association with AUD (15), nicotine dependence (14), and drug dependence (16). In a study conducted by Zuo et al. data for over 26,000 participants were analyzed that included 9 different psychiatric disorders and 15 independent cohorts. All *CHRN* genes, with exception of those that are muscle-type genes, were associated with nicotine dependence and alcohol dependence independently and in participants with both disorders (15). Due to the large sample size and the inclusion of multiple rare variants from the *CHRN* constellation *p*-values were exceptionally strong with *p*-values as low as 10^–39^.

The present study also found variation between probands and controls in eight genes with MAF of 0.001 identified with a Flexible PolyPhen or Flexible REVEL algorithm: *ZNF514*, *OXGR1*, *DIEXF*, *TMX4*, *MTBP*, *PON2*, *ANKRD46*, and *CRHBP*. The possible relevance to AUD is most evident in the case of *CRHBP*, a gene involved in the stress response and a component of the HPA axis. Both animal studies (29) and clinical investigations (34, 35) support the role of HPA axis genes in alcohol consumption. In addition to these eight genes with MAF of 0.001, differences between cases and controls were seen in an additional three genes with MAF of 0.001 with variants that also qualify as protein truncating variants: *AGTRAP*, *ANKRD46*, and *PPA1*. However, no direct association between these genes and alcohol consumption have appeared previously in the clinical or animal literature to our knowledge.

### Analysis of ancestry filtered results

Using the controls filtered to provide a better match to the AUD cases, we found nine genes with statistical significance of 10^–4^. Two of these nine genes were also significant at 10^–4^ in the comparison with the larger unfiltered control set. One gene of special interest, *CHRNA9*, which was significant at 0.0018 in the ancestry controlled comparison was significant at 10^–4^ in the comparison with the larger control sample of 8,983 individuals.

### GWAS rare variants

Gene set analysis was run on 48 genes previously reported by Peng et al. (9) with MAF between 0.03 and 0.005. Four of these genes showed significance at 0.05 in our study. These genes showing marginal significance in our sample are in agreement with the Peng et al. report and appear to hold promise for follow-up.

### Strengths and limitations

The major strength of this study is the sample that was utilized for assessing rare variants. The sample was ascertained using a two proband ascertainment method that required the presence of two same sex siblings with a confirmed diagnosis of AUD to include them and their family members in our family study. All probands and their family members were administered a structured psychiatric interview and diagnoses made using a multimodal approach that included multiple sources of information including results of the structured interview and a follow-up unstructured interview allowing for a best estimate diagnosis to be made. This two proband requirement for inclusion of a family resulted in selection of a set of densely affected families. Such families are ideal for uncovering rare variants. However, this requirement was labor intensive, requiring interviews with approximately 100 potential probands to be able to select one family that met all of the requirements for inclusion. Because the goal was to search for genes conferring greater susceptibility for AUD and provide a sample with minimal comorbidity, families were excluded where a first-degree relative of the proband pair had bipolar disorder, schizophrenia or major depression by DSM criteria.

Inclusion of women with AUD along with the men with AUD from high density families is a strength. An additional strength of the study is the control sample of 8,983 individuals that were sequenced at IGM using the same methodology as the case sample drawn from families with a high density of AUD. Although the control sample contains a wide variety of individuals with varied ethnicity, a secondary analysis was performed utilizing ancestry matched cases and controls that offered additional information. This analysis determined if variants identified in the cases differed from control subjects controlling for ethnic background.

There are limitations in the conclusions that can be drawn based on the small number of cases that could be analyzed though a large set of similarly sequenced controls could be included. Among these is the fact that confirmation in the ancestry filtered analysis could only be performed in a Caucasian (one of the European clusters) set of cases and controls. Analysis of within-family variation using data from all 282 cases is planned and will provide an important confirmation of the strength of conclusions about specific genes. Nevertheless, the genes identified appear to be plausible candidates for future follow-up.

A comment regarding where this report and other rare variant analyses using NGS fits into the search for genetic variation associated with AUD is needed, especially in view of the ever larger GWAS studies currently being published. It has been noted by Povysil et al. (5) that because GWAS searches among common variants, that as sample sizes become ever larger, the newly identified variants have had smaller effects on risk. Additionally, it has been argued that if a sufficient number of individuals are genotyped that the identified variants reported to be associated with complex traits or diseases would be spread broadly and densely across the genome. This omnigenic model though not the intent of individual GWAS studies may have implications for finding mechanistic explanations for disease etiology and medication development. In a review of results for GWAS studies designed to uncover genetic variation for AUD, Hart and Kranzler (47) concluded that findings for GWAS studies of AUD have been largely inconsistent with the exception of variants encoding the alcohol metabolizing enzymes. A large scale meta-analysis of 274,424 individuals (48) combined results of GWAS studies across populations finding 10 variants within genes that appear to confer increased risk for AUD. Among these 10, 4 were variants that encode alcohol metabolizing enzymes. More recently a large scale study of SUD involving over a million participants has been reported (49). This report found some variants common to many specific disorders but others that were unique. Because of the large amount of comorbidity across various SUD including AUD, it may be necessary to focus more attention on acquisition of phenotypically well characterized samples and in the collection of family data where familial comorbidity can be assessed. These steps may reduce the phenotypic heterogeneity making it possible to identify rare variants that provide a mechanistic explanation for the etiology of AUD.

## Data availability statement

The data analyzed in this study is subject to some restrictions. Data were collected before the era of data sharing so consent forms did not include language specifically requesting data sharing. Subsequently, to the extent possible, participants were recontacted and queried regarding sharing. Those willing to share were asked to return a signed consent form. Those willing to share their results allowed us to deposit their data in NIH archives including dbGaP. Data resulting from exome sequencing that were permissible to share are located within the dbGaP repository “Alcohol Dependence Sequencing from Multiplex Families” – https://www.ncbi.nlm.nih.gov/projects/gap/cgi-bin/study.cgi?study_id=phs001775.v1.p1. Further queries can be directed to the corresponding author.

## Ethics statement

The studies involving humans were approved by the University of Pittsburgh Institutional Review Board. The studies were conducted in accordance with the local legislation and institutional requirements. The participants provided their written informed consent to participate in this study.

## Author contributions

SH conceptualized the multiplex design, oversaw the collection of data, and drafted the manuscript. JH collated the appropriate databases, and performed statistical analyses using IGM pipelines under the direction of Dr. David Goldstein, former Director of IGM. Both authors critically reviewed the manuscript providing important feedback for revisions and approved the final submission.

## Footnotes

1
http://genetics.bwh.harvard.edu/pph2/

2
https://github.com/nickzren/atav
