# Supplementary material for: A whole exome sequencing study to identify rare variants in multiplex families with alcohol use disorder
Source: Front Psychiatry. 2023 Oct 17;14:1216493. doi: 10.3389/fpsyt.2023.1216493 (PMC10616827; doi:10.3389/fpsyt.2023.1216493)
Supplement: Supplementary file 1 [file Table_1.docx]

|  |  |  |  |  |  |  |  |  |  |  |  |
| --- | --- | --- | --- | --- | --- | --- | --- | --- | --- | --- | --- |
|  | ***Supplemental Table 1. Odds ratios under varying genetic models comparing probands to all controls.*** | | | | | | | | | |  |
|  |  | ***Qualifying Model*** | ***Qualified***  ***Cases***  ***(N=53)*** | ***Qualified Conrols***  ***(N=8938)*** | | ***Additional***  ***Qualifying Model*** | | | ***Odds Ratio*** | ***Fisher Exact Test p value*** |  |
| ***FleFF***  ***1*** | ***CDSN*** | ***Ultra Rare*** | ***2 (3.77%)*** | ***3 (0.03%*** | |  | | | ***117.39*** | *3.34E-04* |  |
| ***2*** | ***CHRNA9*** | ***Ultra Rare*** | ***2 (3.77%)*** | ***5 (0.06%)*** | | *Flexible PolyPhen* | | | ***70.42*** | *6.96E-04* |  |
| ***3*** | ***IFT43*** | ***Ultra Rare*** | ***2 (3.77%)*** | ***6 (0.07%)*** | |  | | | ***58.67*** | *9.24E-04* |  |
| ***4*** | ***TLR6*** | ***Ultra Rare*** | ***2 (3.77%)*** | ***6 (0.07%)*** | |  | | | ***58.67*** | *9.24E-04* |  |
| *GMPPB* | | *Rare Damaging REVEL* | *3 (5.66%)* | *20 (0.31%)* | | |  | | *26.89* | *3.11E-04* |  |
| *SELENBP1* | | *Rare Damaging REVEL* | *3 (5.66%)* | *28 (0.31%)* | | | *Rare Damaging PolyPhen* | | *19.19* | *7.63E-04* |  |
| *SELENBP1* | | *Rare Damaging PolyPhen* | *3(5.66%)* | *20 (0.22%)* | | | *Rare Damaging REVEL* | | *26.89* | *3.11E-04* |  |
| *ZNF514*   \| *1.22E-04* \| \| --- \| | *Flexible REVEL* | *3(5.66%)* | *14 (0.16%)* | | |  | | *38.44* | *1.22E-04* |  |  |
| *OXGR1* | *Flexible REVEL* | *3(5.66%)* | *23 (0.26%)* | | |  | | *23.37* | *4.50E-04* |  |  |
| *ANKRD46* | *Flexible REVEL* | ***2 (3.77%)*** | *4 (0.04%)* | | |  | | *88.03* | *4.99E-04* |  |  |
| *DIEXF* | *Flexible REVEL* | *3 (5.66%)* | *25 (0.28%)* | | |  | | *21.5* | *5.63E-04* |  |  |
| *TMX4* | | | *Flexible PolyPhen* | *3 (5.66%)* | *12 (0.13%)* | | | *Flexible No Filter* | | *44.86* | *8.25E-05* |
| *MTBP* | | | *Flexible PolyPhen* | *4 (7.55%)* | *65 (0.72%)* | | | *Flexible No Filter* | | *11.2* | *6.88E-04* |
| *PON2* | | | *Flexible PolyPhen* | *3(5.66%)* | *27 (0.30)* | | |  | | *19.9* | *6.92E-04* |
| *CHRNA9* | | | *Flexible PolyPhen* | *4 (7.55%)* | *70 (0.78%)* | | |  | | *10.39* | *8.96E-04* |
| *MTBP* | | | *Flexible No Filter* | *5 (9.43%)* | *103 (1.15%)* | | |  | | *8.98* | *4.04E-04* |
| *TMX4* | | | *Flexible No Filter* | *3(5.66%)* | *25 (0.28%)* | | | *Flexible PolyPhen* | | *21.5* | *5.63E-04* |
| *CRHBP* | | | *Flexible No Filter* | *3(5.66%)* | *26 (0.29%)* | | |  | | *20.67* | *6.25E-04* |
| *AGTRAP* | | | *PTV Loss of Function* | *2 (3.77%)* | *1 (0.01%)* | | |  | | *352.24* | *1.01E-04* |
| *ANKRD46* | | | *PTV Loss of Function* | *2 (3.77%)* | *2 (0,02%)* | | |  | | *176.1* | *2.01E-04* |
| *PPA1* | | | *PTV Loss of Function* | *2 (3.77%)* | *6 (0.07%)* | | |  | | *58.67* | *9.24E-04* |
| *SH3TC2* | | | *Rare Damaging PolyPhen* | *3(5.66%)* | *31(0.35%)* | | |  | | *17.33* | *0.001* |
| *OXGR1* | | | *Flexible PolyPhen* | *3(5.66%)* | *31(0.35%)* | | |  | | *17.33* | *0.001* |
| *IL12RB1* | | | *Rare Damaging REVEL* | *2 (3.77%)* | *10 (0.11%)* | | |  | | *50.29* | *0.0012* |
| *RBM6* | | | *Rare Damaging PolyPhen* | *3(5.66%)* | *33 (0.37%)* | | |  | | *16.27* | *0.0012* |
| *GMPPB* | | | *Flexible REVEL* | *3(5.66%)* | *33 (0.37%)* | | |  | | *16.27* | *0.0012* |
| *VCAN* | | | *Flexible No Filter* | *8 (15.09%)* | *362 (4.03%)* | | |  | | *4.23* | *0.0013* |
| *SH3TC2* | | | *Flexible PolyPhen* | *4 (7.55%)* | *77 (0.86%)* | | |  | | *9.44* | *0.0013* |
| *ASB5* | | | *Flexible PolyPhen* | *3(5.66%)* | *34 (0.38%)* | | |  | | *15.79* | *0.0013* |
| *NGB* | | | *Flexible REVEL* | *2 (3.77%)* | *8 (0.09%)* | | |  | | *44* | *0.0015* |
| *SELENBP1*  *0.0015* | | | *Flexible REVEL* | *3(5.66%)* | *36 (0.40%)* | | |  | | *14.91* | *0.0015* |
| *AGTRAP* | | | *Flexible REVEL* | *2 (3.77%)* | *8 (0.09%)* | | |  | | *44* | *0.0015* |
| *CDSN* | | | *Rare Damaging PolyPhen* | *2 (3.77%)* | *8 (0.09%)* | | |  | | *44* | *0.0015* |
| ***GPI*** | | | ***Ultra Rare*** | *2 (3.77%)* | *8 (0.09%)* | | |  | | *44* | *0.0015* |
| *BRICD5* | | | *Flexible No Filter* | *4 (7.55%)* | *84 (0.94%)* | | |  | | *8.65* | *0.0017* |
| *MYBL1* | | | *Flexible No Filter* | *4 (7.55%)* | *84 (0.94%)* | | |  | | *8.65* | *0.0017* |
| *AKAP11* | | | *Rare Damaing PolyPhen* | *3(5.66%)* | *38 (0.42%)* | | |  | | *14.12* | *0.0017* |
| *IL12RB1* | | | *Flexible PolyPhen* | *3(5.66%)* | *38 (0.42%)* | | |  | | *14.12* | *0.0017* |
| ***PLG*** | | | *Ultra Rare* | *2 (3.77%)* | *9 (0.10%)* | | |  | | *39.1* | *0.0018* |
| ***MAST1*** | | | *Ultra Rare* | *2 (3.77%)* | *9 (0.10%)* | | |  | | *39.1* | *0.0018* |
| *PHF19* | | | *Flexible PolyPhen* | *2 (3.77%)* | *9 (0.10%)* | | |  | | *39.1* | *0.0018* |
| *RAB5B* | | | *Flexible REVEL* | *2 (3.77%)* | *9 (0.10%)* | | |  | | *39.1* | *0.0018* |
| *PHF19* | | | *Flexible REVEL* | *2 (3.77%)* | *9 (0.10%)* | | | *Flexible PolyPhen* | | *39.1* | *0.0018* |
| *OR13G1* | | | *Flexible PolyPhen* | *2 (3.77%)* | *9 (0.10%)* | | |  | | *39.1* | *0.0018* |
| *PPA2* | | | *PTV* | *2 (3.77%)* | *9 (0.10%)* | | |  | | *39.1* | *0.0018* |
| *DOCK11* | | | *Recessive Autosomal* | *4 (8.55%)* | *87 (0.97%)* | | |  | | *8.35* | *0.0019* |
| ***MCF2L2*** | | | *PTV* | *2 (3.77%)* | *10 (0.11%)* | | | *Rare Damaging REVEL* | | *35.19* | *0.0021* |
| *ZHX3* | | | *Rare Damaging REVEL* | *2 (3.77%)* | *10 (0.11%)* | | |  | | *35.19* | *0.0021* |
| *MCF2L2* | | | *Rare Damaging REVEL* | *2 (3.77%)* | *11 (0.12%)* | | |  | | *31.99* | *0.0025* |
| *IFT43* | | | *Rare Damaging PolyPhen* | *2 (3.77%)* | *11 (0.12%)* | | |  | | *31.99* | *0.0025* |
| *HEXDC* | | | *Rare Damaging PolyPhen* | *2 (3.77%)* | *11 (0.12%)* | | |  | | *31.99* | *0.0025* |
| *CCL22* | | | *Flexible No Filter* | *2 (3.77%)* | *11 (0.12%)* | | |  | | *31.99* | *0.0025* |
| ***CDH16*** | | | *Ultra Rare* | *2 (3.77%)* | *11 (0.12%)* | | |  | | *31.99* | *0.0025* |
| *RNF32* | | | *Flexible No Filter* | *3 (5.66%)* | *44 (0.49%)* | | |  | | *12.19* | *0.0026* |
| *PON2* | | | *Flexible No Filter* | *3 (5.66%)* | *44 (0.49%)* | | |  | | *12.19* | *0.0026* |
| *NUP54* | | | *Flexible No Filter* | *3 (5.66%)* | *44 (0.49%)* | | |  | | *12.19* | *0.0026* |
| *BDP1* | | | *Recessive Autosomal* | *3 (5.66%)* | *44 (0.49%)* | | |  | | *12.19* | *0.0026* |
| *POLR3A* | | | *Rare Damaging REVEL* | *3 (5.66%)* | *46 (0.51%)* | | |  | | *11.66* | *0.0029* |
| *GORAB* | | | *Rare Damaging PolyPhen* | *2 (3.77%)* | *12 (0.13%)* | | |  | | *29.32* | *0.0029* |
| *NDUFB8* | | | *Rare Damaging PolyPhen* | *2 (3.77%)* | *12 (0.13%)* | | |  | | *29.32* | *0.0029* |
| *FANCA* | | | *PTV Loss of Function* | *2 (3.77%)* | *13 (0.14%)* | | |  | | *27.06* | *0.0034* |
| *SMPD2* | | | *Rare Damaging REVEL* | *2 (3.77%)* | *14 (0.16%)* | | |  | | *27.06* | *0.0034* |
| *TLR6* | | | *Rare Damaging PolyPhen* | *2 (3.77%)* | *13 (0.14%)* | | |  | | *27.06* | *0.0034* |
| *PON2* | | | *Rare Damaging REVEL* | *2 (3.77%)* | *14 (0.16%)* | | |  | | *25.12* | *0.0038* |
| ***ZHX3*** | | | *Ultra Rare* | *2 (3.77%)* | *14 (0.16%)* | | |  | | *25.12* | *0.0038* |
| *SLC34A3* | | | *Rare Damaging REVEL* | *2 (3.77%)* | *16 (0.18%)* | | |  | | *21.98* | *0.0049* |
| *RUFY1* | | | *Rare Damaging REVEL* | *2 (3.77%)* | *16 (0.18%)* | | |  | | \| \| \| \| *21.98* \| *21.98* \| \| --- \| --- \| \| *21.98* \| \| --- \| --- \| --- \| --- \| \| *21.98* \| \| --- \| --- \| --- \| --- \| --- \| --- \| \| *21.98* \| \| --- \| --- \| --- \| --- \| --- \| --- \| --- \| --- \| | *0.0049* |
| *AMZ1* | | | *PTV Loss of Function* | *2 (3.77%)* | *16 (0.18%)* | | |  | | *21.98* | *0.0049* |
| *ITPR2* | | | *PTV Loss of Function* | *2 (3.77%)* | *17 (0.19%)* | | |  | | *20.68* | *0.0054* |
| *MUC16* | | | *Recessive Autosomal* | *0 (0.00%)* | *956 (10.64* | | |  | | *0* | *0.0055* |
| *GPR119* | | | *PTV Loss of Function* | *1 (1.89%)* | *0 (0.00)* | | |  | | *NA* | *0.0059* |
| *PTF1A* | | | *PTV Loss of Function* | *1 (1.89%)* | *0 (0.00)* | | |  | | *NA* | *0.0059* |
| *OSER1* | | | *Recessive Autosomal* | *1 (1.89%)* | *0 (0.00)* | | |  | | *NA* | *0.0059* |
| *CIB3* | | | *Recessive Autosoma* | *1 (1.89%)* | *0 (0.00)* | | |  | | *NA* | *0.0059* |
| *CSRP3* | | | *Recessive Autosoma* | *1 (1.89%)* | *0 (0.00)* | | |  | | *NA* | *0.0059* |
| *NUDCD3* | | | *Recessive Autosoma* | *1 (1.89%)* | *0 (0.00)* | | |  | | *NA* | *0.0059* |
| *TRPC1* | *Recessive Autosoma* | *1 (1.89%)* | | *0 (0.00)* | | |  | *NA* | *0.0059* |  |  |
| *HEPACAM2* | *Recessive Autosoma* | *1 (1.89%)* | |  | | |  | *NA* | *0.0059* |  |  |
| *C12orf49* | *Recessive Autosoma* | *1 (1.89%)* | |  | | |  | *NA* | *0.0059* |  |  |
