# Supplementary material for: A whole exome sequencing study to identify rare variants in multiplex families with alcohol use disorder
Source: Front Psychiatry. 2023 Oct 17;14:1216493. doi: 10.3389/fpsyt.2023.1216493 (PMC10616827; doi:10.3389/fpsyt.2023.1216493)
Supplement: Supplementary file 2 [file Table_2.docx]

|  | ***Supplemental Table 2. Odds ratios under varying genetic models comparing probands with European ancestry to a subset of controls with European ancestry.*** | | | | | | | | |  |
| --- | --- | --- | --- | --- | --- | --- | --- | --- | --- | --- |
|  |  | ***Qualifying Model*** | ***Qualified***  ***Cases***  ***(N=52)*** | ***Qualified Conrols***  ***(N=2841)*** | ***Additional***  ***Qualifying Model*** | | ***Additional Qualifying Model*** | ***Odds Ratio*** | ***Fisher Exact Test p value*** |  |
| ***FleFF***  ***1*** | *ADCY10* | ***PTV, Ultra Rare LOFTEE*** | ***2 (3.85%)*** | ***1 (0.04%)*** | ***Ensemble Ultra Rare*** | |  | ***112.52*** | ***9.58 X 10^-4^*** |  |
| ***2*** | *KIAA0513* | ***PTV, Ultra Rare LOFTEE*** | ***2 (3.85%)*** | ***1 (0.04%)*** | ***Ensemble Ultra Rare*** | |  | ***112.52*** | ***9.58 X 10^-4^*** |  |
| ***3*** | *MCF2L2* | ***PTV, Ultra Rare LOFTEE*** | ***2 (3.85%)*** | ***2 (0.07%)*** |  | |  | ***56.24*** | ***0.001893*** |  |
| ***4*** | *GORAB* | ***PTV LOFTEE*** | ***2 (3.85%)*** | ***0 (0.00%)*** |  | |  | ***NA*** |  |  |
| *MTBP* | | ***PTV LOFTEE*** | ***2 (3.85%)*** | \| ***1 (0.04%)*** \| \| --- \| \| ***1 (0.04%)*** \| | |  |  | *112.52* | ***9.58 X 10^-4^*** |  |
| *KIAA0513* | | ***PTV LOFTEE*** | ***2 (3.85%)*** | \| ***1 (0.04%)*** \| \| --- \| \| ***1 (0.04%)*** \| | |  |  | *112.52* | ***9.58 X 10^-4^*** |  |
| *RUFY1* | | *Ensemble Ultra-Rare* | ***2 (3.85%)*** | ***0 (0.00%)*** | | *Ensemble Rare*  *1.08* ***X 10^-4^*** | *AF =0.1% and 1%* | *112.52* | ***9.58 X 10^-4^*** |  |
| *ZHX3* | *Ensemble Ultra-Rare* | ***2 (3.85%)*** | ***1 (0.04%)*** | |  |  | *112.52* | ***9.58 X 10^-4^*** |  |  |
| *GPI* | *Ensemble Ultra-Rare* | ***2 (3.85%)*** | ***1 (0.04%)*** | |  |  | *112.52* | ***9.58 X 10^-4^*** |  |  |
| *KIAA0513* | *Ensemble Ultra-Rare* | ***2 (3.85%)*** | ***1 (0.04%)*** | |  |  | *112.52* | ***9.58 X 10^-4^*** |  |  |
| *ADCY10* | *Ensemble Ultra-Rare* | ***2 (3.85%)*** | ***1 (0.04%)*** | |  |  | *112.52* | ***9.58 X 10^-4^*** |  |  |
| *MAN2A2* | | | *Ensemble Ultra-Rare* | ***2 (3.85%)*** | *2 (0.07%)* | |  |  | *56.24* | *0.001893* |
| *PDLR3A* | | | *Ensemble Ultra-Rare* | ***2 (3.85%)*** | *2 (0.07%)* | | *AF=0.1% and AF=1%* |  | *56.24* | *0.001893* |
| *CHRNA9* | | | *Ensemble Ultra-Rare* | ***2 (3.85%)*** | *2 (0.07%)* | |  |  | *56.24* | *0.001893* |
| *AGTRAP* | | | *Ensemble Filter 0.1%* | *2****(3.85%)*** | ***1 (0.04%)*** | | *AF=1%* |  | *112.52* | ***9.58 X 10^-4^*** |
| *COLBA2* | | | *Ensemble Filter 0.1%* | *2****(3.85%)*** | ***1 (0.04%)*** | | *AF=1%* |  | *112.52* | ***9.58 X 10^-4^*** |
| *DAGLB* | | | *Ensemble Filter 0.1%* | *2****(3.85%)*** | ***1 (0.04%)*** | | *AF=1%* |  | *112.52* | ***9.58 X 10^-4^*** |
| *5H3TC2* | | | *Ensemble Filter 0.1%* | *3 (5.77%)* | *9 (0.32%)* | | *AF=1%* |  | *19.08* | *0.001105* |
| *MZF1* | | | *Ensemble Filter 0.1%* | *3 (5.77%)* | *11 (9.39%)* | | *AF=1%* |  | *15.6* | *0.0011781* |
| *ZHX3* | | | *Esemble Filter 0.1%* | *2****(3.85%)*** | *2 (0.07%)* | |  |  | *56.24* | *0.001893* |
|  | | |  |  |  | |  |  |  |  |
|  | | |  |  |  | |  |  |  |  |
|  | | |  |  |  | |  |  |  |  |
|  | | |  |  |  | |  |  |  |  |
